# Dosimetry and Treatment Descriptions Using the First Completely Automated Stereotactic Intracranial Radiosurgery Rotating Gamma Ray Unit in America

**DOI:** 10.7759/cureus.2542

**Published:** 2018-04-27

**Authors:** Eduardo E Lovo, Fidel J Campos, Victor E Caceros, Mario H Minervini, William A Reyes

**Affiliations:** 1 Radiosurgery Program, Centro Internacional De Cancer, Hospital De Diagnostico, San Salvador, SLV

**Keywords:** radiosurgery, stereotactic radiosurgery, gamma-knife, brain, dosimetric

## Abstract

Introduction

The objective is to report the dosimetry and safety profiles of the first fully automatized rotating gamma ray unit for intracranial radiosurgery in America.

Methods

Dosimetry tests were conducted by our institution using the standard of examination and calibration and the Intelligent γ Radiometer of the China Research Institute of Measurement. The phantom and dosimetry tests were performed by the Outreach Physics Section of MD Anderson Cancer Center and the Anchorage Radiation Therapy Center using the Radiation Therapy Oncology Group (RTOG) radiosurgery quality assurance guidelines. Clinically, 233 patients were treated.

Results

Mechanical precision was 0.16 mm and the offset registered at the phantom on all axes was 0.0. The ratio of the dose to the center was 0.97 (0.95-1.05), the ratio of the treated volume was 0.95 (0.75-1.25), the ratio of the measured treated volume to the volume of the target was 1.29 (1.00-2.00), the ratio of the minimal dose to the dose prescribed was 1.05 (>0.90), with a treated volume of 0.95 (0.75-1.25) and a minimum dose to target of 1.05 (>0.90). The dose rate at loading was 3.89 Gy per minute. None of the patients treated experienced severe complications.

Conclusions

The dosimetry studies are compliant with quality assurance standards for intracranial radiosurgery.

## Introduction

In accordance with the American Society for Therapeutic Radiology and Oncology (ASTRO) and others, part of the definition of stereotactic radiosurgery is, “it is a discipline that utilizes externally generated ionizing radiation to inactivate or eradicate (a) defined target(s) in the head or spine without the need to make an incision, while defining the target by high-resolution imaging” [1].

Intracranial stereotactic radiosurgery was developed by Lars Leksell and the tool he and his group developed: the Gamma knife. Since 1968, this has been demonstrated to be extremely precise (<1 mm) and capable of producing a steep dose gradient of radiation that is, till date, the gold standard and to what, typically, other radiosurgery systems compare to [2]. In the evolution of this technology, the amount of cobalt 60 sources reduced slightly from 201 in model U to 192 in Perfexion (Elekta Instruments AB, Stockholm, Sweden). The main advances have been automation as well as intracranial reach, which increased dramatically according to some authors [3]. Image guidance is also available now, which allows multisession radiosurgery.

During the 1990s, other companies in Asia begun developing intracranial stereotactic gamma ray machines that rotated the cobalt 60 sources in relation to the isocenter, needing a significantly lower number of sources (25 or 30) with similar dosimetry characteristics than the static Gamma knife units [4-5] of the time. In 2014, the first fully automated rotating gamma ray unit called Infini (Masep Medical Company, Shenzhen, China) came into service in the American continent and 233 patients have been treated so far.

The purpose of the current communication is to report the basic dosimetry and patient treatment process to better understand this relatively novel intracranial stereotactic radiosurgery machine.

## Materials and methods

The current investigation was carried out from March 2014 to January 2017. During this period, 30 cobalt 60 sources were loaded on a fully automated, rotating gamma-ray system called Infini. Dosimetry studies were carried out by the institution where Infini was installed and three outside institutions participated in a phantom test analysis before the first patient was treated. During this time, 233 patients were treated for different pathological entities affecting the brain.

Machine characteristics

**Figure 1:**
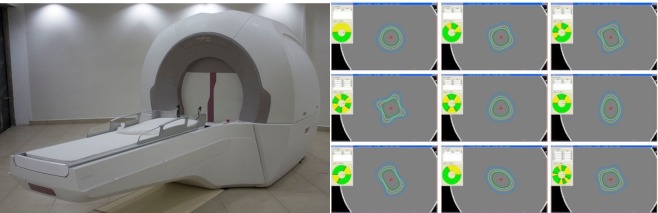
View of Infini and some dose sculpturing capabilities Composite image: To the left is an oblique view of Infini (Masep Medical Company, Shenzhen, China); to the right are different dose configurations that can be achieved by selectively blocking different sectors at 5 degrees or more.

Infini is a fully automated, rotating gamma-ray machine designed for intracranial stereotactic radiosurgery (Figure 1). It uses 30 rotating cobalt 60 sources that revolve with regards to the mechanical isocenter using a multisource, non-isometric spiral focusing method. These cobalt sources are arranged in six rows of five cobalt sources each and rotate at a one rotation per minute speed. It has four different sizes of collimators (4, 8, 14, 18 mm) to choose from, as well as three different angles that can be used, 70, 90, and 110°, in accordance with the head holder and the trunnion of the machine. The intracranial reach is 180/180/230 mm in the X/Y/Z axis, respectively. The operations of the collimators and patient positioning are completely automated, allowing a seamless workflow. Finally, it has an independent beam switch that can turn on or off the passage of radiation every 5 degrees.

Dosimetry evaluation process

Dosimetry results were obtained from final commissioning by the local institution and the manufacturing company using the standard of examination and calibration of the Intelligent γ Radiometer of the China Research Institute of Measurement. Also, a stereotactic radiosurgery head phantom irradiation test was performed and audited by the Outreach Physics Section of Imaging and Radiation Oncology Core (IROC), an institution that belongs to MD Anderson Cancer Center (Houston, TX, USA), as follows: An anthropomorphic head phantom with a 1.9 cm diameter spherical target was mounted on the stereotactic frame and was irradiated with a single isocenter with a maximum dose to the target of 30 Gy. The doses to two specific points were measured using thermoluminescent dosimetry (TLD) capsules and the dose distribution was evaluated using two orthogonal sheets of radiochromic film. 

The dose rate was confirmed by the Anchorage Radiation Therapy Center by irradiation and taking readings from the center of the 16 cm polystyrene phantom using an 18 mm collimator utilizing the TG-21 American Association of Physicist in Medicine (AAPM) protocol to determine the dose rate. The dosimetry equipment used was an ionization chamber, Exradin (A1SL SN XW 060580) and an electrometer, Standard Imaging MAX-4000 (SN 131014).

Patient treatment flow

At the time of this report, 233 patients had been treated with diverse intracranial lesions and neurological and psychiatric conditions (Table 1). In brief, the process regarding patient treatment is as follows: The patient is admitted by an anesthesiologist that provides slight sedation using intravenous fentanyl, as needed. At the same time, 8 mg of decadron (Dexamethasone) is administered intravenously. The stereotactic frame is placed while the patient is sedated under local anesthesia using a mixture of 50/50 lidocaine and bupivacaine at the sites where the pins will pierce the skin into the external bone cortex of the cranium. Immediately after the frame has been secured to the patient, the N reference box is fixed to the frame and an MR head holder adaptor and antenna are fixed in the MRI unit, Avanto 1.5 Tesla (Siemens Corporation, Erlangen, Germany). The following sequences are carried out: one T2, 6-mm axial sequence at 0⁰ of the full head, which is later used in the Superplan (Masep Medical Co., Shenzhen, China) treatment planning system (TPS) to provide the volume of the patients' skin, one T1-gadolinium 1-mm axial and one coronal sequence of the area of interest at 0⁰ and 90⁰ respectively is carried out in cases where tumors need to be identified. In cases of metastasis, the T1 is done with a double dose of gadolinium for the entire head and acquired at 1-mm axial slices. When AVMs are being treated, we also use an axial T2 CISS of 1 mm of the region of interest in addition to the T1-gadolinium. The same sequence is used when detail is needed of the intracranial nerves. When functional targets inside the brain are being chosen, a T1 without gadolinium or CISS and fluid attenuation inversion recovery (FLAIR) sequences with 1-mm slice widths are used to visualize internal capsules or other structures, such as anterior and posterior commissures. All sequences required a field of view (FOV) of 240 for proper visualization on the TPS. More recently, we performed image fusion (two stereotactic image sets fused using the reference box marks as guides) with CT and used this as the primary study in cranium base tumors or patients where an MRI is not feasible when patients are overweight or have pacemakers.

**Table 1:** Pathology and treatment characteristics Treatment characteristic of the whole series shown by general classifications of pathologies, patient characteristics and mean marginal dose. * IDL- Isodose lines

Pathology	Number of treatments	Age	Gender	Mean Marginal dose
Metastasis and malignant glioma	41 (17.5%)	57.6 (7-78)	119 (51%) Female 114 (49%) Male. For whole series	21 Gy (10-24 Gy) to the 56% IDL* (49-90%)
Benign tumor	97 (41.6%)	52 (28-76)		14 Gy (10-22 Gy) to the 50% IDL (49-52%)
Vascular pathology	43 (18.3%)	26 (16-67)		18 Gy (16-20 Gy) to the 50% IDL
Functional	52 (22.3%)	59 (31-80)		100 Gy (75-150 Gy) to the 100% IDL

The images were transferred to TPS via the ethernet. Regions of interest (ROI), as well as the Planned Target Volume (PTV  ) or target, are generally contoured by the neurosurgeon in collaboration with the radiation oncologist. The final plan is configured by the physicist with the participation of the neurosurgeon and radiation oncologist. Once it has been approved, the patient is carried into the Infini and fixed to the treatment bed with the head frame holder to the trunnion on the treatment couch and the angle of treatment is verified so that the irradiation can begin. After the treatment is finished, the frame is removed from the patient and they are instructed to remove the circular band-aids placed at the pin sites 24 hours after the treatment. They are discharged immediately after treatment.

## Results

The mechanical precision of the machine was 0.16 mm, on average, for all the collimators tested (0.11 for the 4 mm, 0.16 for the 8 mm, 0.17 for the 14 mm, and 0.20 for the 18 mm). The offset registered at the head phantom irradiation test by MDACC with a prescription dose of 15 Gy to the 50% isodose line on all axes was 0.0, as measured along the different profiles.

**Figure 2:**
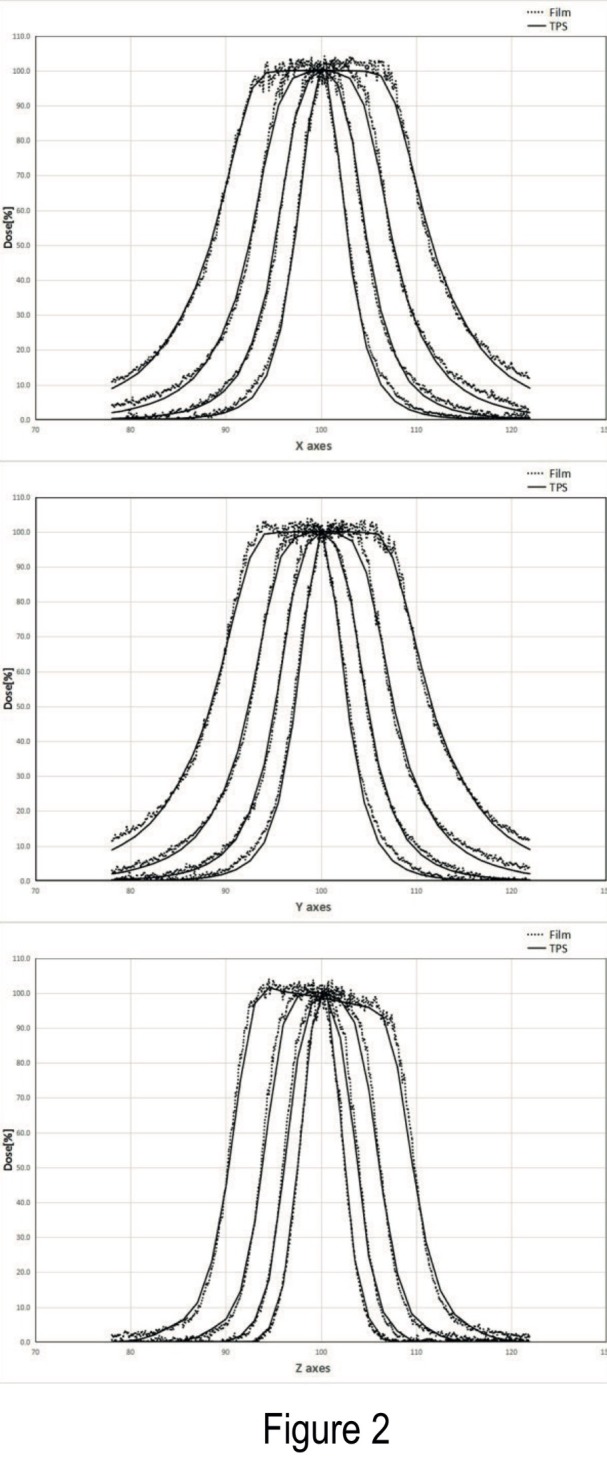
Beam profiles for different collimator sizes Profiles calculated from different-sized collimators - 4, 8, 14, and 18 mm - for all stereotactic X, Y, and Z axes. On top is the X axis, in the middle is the Y axis, and at the bottom of the image is the Z axis.

The precision of beam alignment is determined by comparing the measured dose profiles of the resulting dose distribution located in a volume surrounding the radiological focus point with the calculated dose profiles, assuming identical geometrical and radio physical conditions. An acceptance criterion with a deviation between dose profiles of no more than –+1 mm at the 50% isodose line, it was accomplished with all the dose profiles in accordance to manufacturer specifications (Figure 2).

The initial dose rate at the isocenter was 3.89 Gy per minute using the 18 mm collimator. The results of the head anthropomorphic phantom irradiation test were the following: The ratio of the dose to the center of the target measured by IROC to the dose reported by the local institution was 0.97 (acceptance criteria: 0.95-1.05), the ratio of treated volume taken from the films' evaluation was 0.95 (acceptance criteria: 0.75-1.25), the ratio of the measured treated volume to the volume of the target was 1.29 (acceptance criteria: 1.00-2.00), and, finally, the ratio of the minimal dose to the dose prescribed was 1.05 (acceptance criteria >0.90), with a treated volume of 0.95 (criteria: 0.75-1.25) with a minimum dose to target of 1.05 (criteria >0.90).

**Figure 3:**
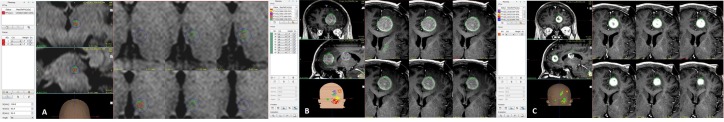
Examples of clinical applications A. Dosimetry and isodose lines of a medial thalamotomy plan for intractable contralateral pain from trigeminal neuralgia. The inner isodose line represents the 75% curve of the total dose, the middle isodose line represents the 50%, and the outer isodose line represents 25% of 140 Gy of the prescription dose. B. and C. are two different treatments plans for a large cerebral metastasis spaced by 30 days in a protocol for dose escalation. In each treatment, a prescription dose of 1350 cGy to the 50% and 53% isodose lines was used, respectively, with a median dose of 1942 cGy per treatment.

Patient and treatment characteristics can be seen in Table 1. Thus far, out of the 233 patients treated, most were female - 119 (51%) with 114 male (49%), the median age was 50.3 years (7-80), 138 patients (59.2%) were treated for malignant or benign tumors, 43 patients (18.3%) were treated for vascular pathology, and 52 others (22%) were treated under functional radiosurgery protocols for trigeminal neuralgia, thalamotomy, hypophysectomy, and anterior capsulotomy (Figure 3).

The mean follow-up was 26.2 months (4-39) and only two cases (0.8%) experienced  Radiation Therapy Oncology Group (RTOG) toxicity exceeding grade 1; both were in brain metastases cases caused by radiation necrosis.

## Discussion

Although the mechanical precision of older models of rotating gamma ray units had already been evaluated in comparison to the gamma knife models of that time and deemed similar [4-5], this is the first report of the radiological accuracy of a fully automatized rotating gamma ray unit known as Infini. Since this model is a relatively novel intracranial stereotactic radiosurgery unit and few exist in the world at this moment, it is necessary to report from the dosimetry point of view that it is compliant with regards to precision and beam irradiation characteristics with the RTOG guidelines [6].

As demonstrated by dosimetry results, Infini is comparable to the Perfexion and older versions of gamma knife. One difference is the higher initial cobalt 60 activity (2.4x10₁₄Bq) at the loading of the Infini that gave it a greater initial dose rate (3.89 Gy per minute) than the one reported by Perfexion [7]; thus, possibly traducing in faster treatment times despite the small amount of cobalt 60 sources. One of the clearest advantages of the rotating principle is the need for a small number of cobalt sources (30 vs. 192) that transduces into lower costs and higher efficiency. This aspect was very important for underdeveloped countries such as the one where the system was installed.

At the time of this report, over 233 patients have been treated. Although it is not the intention of this report to evaluate the clinical results of the diverse pathologies treated, enough follow-up has been done to say that the treatment is safe and has low complications associated with the procedure, even under extreme uses, such as functional diseases where very high doses of radiation are delivered to small areas in very eloquent regions of the brain.

## Conclusions

Infini is a completely automatized intracranial stereotactic machine that uses 30 cobalt sources in an array that circulates around the isocenter and thus the target. The dosimetry studies and clinical use has demonstrated that it is safe and compliant with the RTOG quality assurance standards for intracranial radiosurgery.
